# Sprouty1 exerts a preventive effect on the initiation of psoriasis by inhibiting innate immune antimicrobial peptide cathelicidin and immunocytes

**DOI:** 10.1111/cpr.13290

**Published:** 2022-06-18

**Authors:** Yuan Zhou, Ping Wang, Xue‐Yan Chen, Bing‐Xi Yan, Lilla Landeck, Zhao‐Yuan Wang, Fan Xu, Min Zheng, Xiao‐Yong Man

**Affiliations:** ^1^ Department of Dermatology Second Affiliated Hospital, Zhejiang University School of Medicine Hangzhou China; ^2^ Department of Dermatology Ernst von Bergmann General Hospital Potsdam Germany

## Abstract

**Objectives:**

Psoriasis is an immune‐mediated skin disease dominated by the cutaneous immune system. Keratinocytes have been considered important triggers that initiate psoriasis. The key molecules and events of keratinocytes that link the innate immune system in psoriasis must be investigated in more detail. Human psoriasis skin and primary human keratinocyte were detected in vitro. Epidermis specific transgenic mouse strain (Krt14‐Sprouty1 tg) was used to further investigate psoriasis‐like skin inflammation in vivo.

**Materials and Methods:**

Bulk RNA sequencing of primary human keratinocyte screened differentially expressed genes, which was confirmed by quantitative real time PCR and Western Blot (WB). Moreover, we concomitantly reviewed open‐accessed published RNAseq datasets of human psoriatic skin from GEO database. Immunohistochemical staining and immunofluorescence were used to detect Sprouty1 (SPRY1) expression in human psoriatic skin with and without anti‐psoriasis treatments. Krt14‐Sprouty1 tg was used to further investigate psoriasis‐like skin inflammation, and followed by Hematoxylin and Eosin (HE) Staining, enzyme linked immunosorbent assay (ELISA), Western Blot and flow cytometry.

**Results:**

Our data showed that Sprouty1 was decreased in psoriatic skin and keratinocytes. In imiquimod‐induced psoriasis‐like skin inflammation, the production of cathelicidin (camp/LL37) was inhibited by suppressing signal transducer and activator of transcription3 (Stat3) activation when Sprouty1 overexpressed in mouse epidermal keratinocytes. Moreover, CD11b+CCR2+ dendritic cells, IL‐17A+ γδT cells, and Ly6C+ CD11c+ monocyte‐derived dendritic cells were decreased in Krt14‐Sprouty1 tg (STG) imiquimod‐induced cutaneous inflammation.

**Conclusions:**

These findings indicate that Sprouty1 expressed in keratinocytes has a suppressive role in imiquimod‐induced skin inflammation mediated by inhibiting the production of cathelicidin. Collectively, Sprouty1 plays a preventive role in psoriatic skin. Our data provide new evidence for the pathogenesis of psoriatic keratinocytes, and the link cutaneous innate immunity, that indicated Sprouty1 is a potential novel therapeutic target.

## Graphical abstract

Sprouty1 can inhibit innate immune antimicrobial peptide cathelicidin (LL37) by suppressing Stat3 activation in psoriatic keratinocytes, which not only inhibit epidermal langerhans cells and dendritic cells but also suppress the recruitment of dermal γδT cells and monocyte‐derived dendritic cells infiltration in imiquimod‐induced skin inflammation.

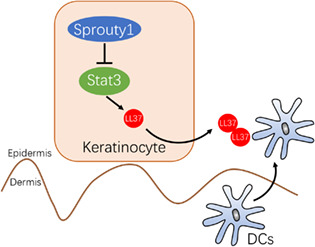

## 1 INTRODUCTION

Psoriasis is a clinically heterogeneous immune‐mediated disease that is characterized by circumscribed large, silvery scaling and erythematous plaques.^1^ Epidermal hyperplasia, angiogenesis and leukocyte infiltration of both the dermis and epidermis are common histologic characteristics of psoriasis.^1^, ^2^, ^3^, ^4^ In the past, this typical epidermal involvement has led to discussions on whether hyperproliferation and altered differentiation of epidermal keratinocytes occurs only in response to skin inflammation or whether keratinocytes themselves are partly responsible for the initiation and/or propagation of psoriasis.^5^, ^6^, ^7^, ^8^ Besides keratinocyte initiation, IL‐23/ suppressing signal transducer and activator of transcription3 (STAT3) pathways also are key player development and pathogenesis of psoriasis,^9^ and Janus kinase (JAK)/STAT3 are potential psoriasis therapeutic target.^10^, ^11^

Sprouty (SPRY) proteins include four orthologs (SPRY1, 2, 3 and 4), which modulate a receptor tyrosine kinase (RTK)/mitogen‐activated protein kinase (MAPK)‐induced signalling pathway. Sprouty1, as known as RTK signalling antagonist 1, inhibits signalling through various growth factor pathways and has also been shown to be a tumour suppressor in various malignancies.^12^ Our previously published data demonstrated an important impact of Sprouty1 on the biological functions of keratinocytes, including inhibiting proliferation, promoting apoptosis, and regulating differentiation. These findings were confirmed in epidermis‐specific Krt14‐SPRY1 transgenic mice.^12^ Moreover, we found that Sprouty1 was decreased in psoriatic skin and keratinocytes by bulk RNA sequencing, quantitative real‐time PCR and Western blotting. Recently, other studies showed that SPRY1 could influence the survival and exhaustion of T cells.^13^, ^14^ Hypothetically, SPRY1 may be a negative feedback regulator in cutaneous inflammatory responses. Based on these observations, we investigated the role of SPRY1 in the construction of the skin barrier and the regulation of the innate immune system, especially in human psoriatic skin.

## 2 MATERIALS AND METHODS

### 2.1 Patients

Subjects were recruited at Second Affiliated Hospital, Zhejiang University School of Medicine. The study was approved by the Institutional Review Board of Second Affiliated Hospital, Zhejiang University School of Medicine. Written and informed consent was obtained from all psoriasis patients and healthy controls (details shown in Table S1).

### 2.2 Mouse strains

C57BL/6J wild‐type (WT) mouse strain was purchased from Slaccas Animal Research Center (Shanghai, China). Krt14‐hSPRY1 transgenic (Krt14‐SPRY1‐tg, STG) mice were previously generated on C57BL/6 background by Suzhou cyagen company. All animal studies were approved by the Animal Care and Use Committee, Second Affiliated Hospital, Zhejiang University School of Medicine. All experiments were conducted in accordance with the institutional ethical guidelines for the care and use of laboratory animals. Mice were bred and maintained under specific‐pathogen‐free conditions at the Animal Center, Zhejiang Traditional Chinese Medicine University and housed in accordance with the procedures outlined in the Guide for the Care and Use of Laboratory Animals. Animal studies were not performed in a blinded fashion. Each mouse was of same gender, whose weight was between 18 and 20 g, and was assigned randomly to experimental groups by a random digit table. The number of animals is shown in each figure.

### 2.3 Isolation and culture of primary human and mouse epidermal keratinocytes

Normal human epidermal keratinocytes (NHEK) and human primary psoriatic epidermal keratinocytes (PLEK) were prepared from skin incubated with dispase as previously described.^15^ Briefly, skin specimens were incubated with 0.5% dispase overnight at 4°C. Epidermal and dermal sheets were separated and placed. Second passage with the confluence status of NHEK and PLEK was used in this study.

### 2.4 Bulk RNA sequence and quantitative real‐time PCR

Primary cultured human epidermal keratinocytes and mouse skin were lysed by TRIzol reagent (Invitrogen) followed by protocol. Bulk RNA sequences were performed in Illumina Platform by the Beijing Genomics institution (BGI, China). Quantitative real‐time PCR was done according to the previous description.^12^ CT values were analysed by qBase software (Biogazelle, Zwijnaarde, Belgium).

### 2.5 RNA sequence reanalysis from Gene Expression Omnibus

Open‐accessed published RNAseq datasets of human psoriatic skin from Gene Expression Omnibus (GEO), GEO GDS4602, GDS3539, GDS5420 and GDS4606, were referred to in this paper. The expression of Sprouty1 was downloaded from GEO, and represented by Graphpad.

### 2.6 Western blot

Cultured cells and mouse epidermis were lysed with radioimmunoprecipitation assay buffer and lysates were boiled with 5 × loading buffer for 10 min. The samples were then separated with SDS‐PAGE gel and immunoblotted with indicated antibodies (Table S2), followed by an incubation of a second antibody. The immunoreactive bands were detected by using ECL Substrate (Thermo Scientific™).

### 2.7 Immunohistochemistry, immunofluorescence and haematoxylin and eosin staining

For immunohistochemistry of SPRY1, optimal cutting temperature compound embedded sections were used. Slides were then blocked with normal rabbit or goat serum (Vector Laboratories, CA) and incubated with primary antibodies (Abcam) followed by incubation with biotinylated rabbit anti‐rat IgG or biotinylated goat anti‐rabbit IgG. Staining was finally visualized with DAB high‐sensitivity substrate chromogen solution (Vector Laboratories, CA) and counterstained with haematoxylin. An immunofluorescence assay was carried out as described in detail before.^12^ Briefly, after incubation with primary antibody, skin biopsy specimens were incubated with secondary antibodies, Alexa Fluor™ 488 and 555 (Invitrogen). Nuclei were counterstained with DAPI. For each case, a negative control incubated with non‐immune mouse IgG (Sigma‐Aldrich) was included. The sections of Mouse skin were stained with haematoxylin and eosin after being sectioned by a freezing microtome (Leica).

### 2.8 Psoriasis‐like skin inflammation models and siRNA injection

The dorsal skin of 8‐week‐old mice in the telogen phase of the hair cycle was shaved with clippers and then subjected to topical application of imiquimod on the skin. Mice were treated with either ~1 mg/cm^2^ skin of 5% Imiquimod (IMQ) cream (Perrigo) or vehicle as a control for five consecutive days as previously described.^15^ 2′‐oMe modified Camp‐siRNA were synthesized by Genomeditech (Shanghai, China), 5 nM/20 g mouse, whose sequence(5′‐3′): CAGCCCUUUCGGUUCAAGAAA tt, UUUCUUGAACCGAAAGGGCUG tt.

### 2.9 Extraction of mice epidermal protein

The mouse skin tissues were cut after different treatments, immediately placed in 4–6 ml 0.5% dispase and incubated overnight at 4°C. The next day, the epidermis and dermis were separated. The epidermis was placed in a cooled mortar and an appropriate amount of liquid nitrogen was added to the grind. After the tissue is grounded to powder, an appropriate amount of protein lysis solution was added by pipetting and mixing, and the lysis solution was collected in an EP tube and then lysed on ice for 20 min, 14,000 rpm, 4°C centrifugation, 10 min. The subsequent processing method was the same as the preparation of cell protein samples.

### 2.10 The enzyme‐linked immunosorbent assay

The mouse epidermis protein samples were incubated in a pre‐coated mouse LL37 (Cathelicidin) enzyme‐linked immunosorbent assay (ELISA) plate (LSBio) for 1 h at 37°C. The detected antibody was added and incubated for 1 h at 37°C. Then it was washed three times, followed by incubation with Streptavidin‐HRP Complex for 30 min at 37°C. After adding TMB substrate, we then terminated with a stop solution. The optical density value was determined by immediately using a reader at 450 nm (BioTek, ELx808).

### 2.11 Preparation of skin tissue single‐cell suspension

The mouse skin tissue was incubated overnight in the dispase enzyme at 4°C. Then it was carefully separated from the epidermis and dermis, and the separated epidermis and dermal tissue were cut into different culture dishes. A digestion solution was subsequently added and mixed by pipetting. The samples were thus placed in a 37°C incubator for 30 min and filtered with a 200 cell mesh. The digestion solution was collected in a centrifuge tube. Centrifugation was performed at 1000 rpm for 5 min, the supernatant was discarded, 2 ml PBS was added to resuspend the pellet, and the second cycle of centrifugation at 1500 rpm for 5 min was performed. To obtain a single‐cell suspension, samples were grouped according to flow cytometry, the cells were resuspended in 100 μl PBS for each group, and incubated according to the recommended concentration of different flow cytometry antibodies.

### 2.12 Flow cytometric analysis and cell sorting

The following Abs were purchased from Biolegend. To stain intracellular cytokines, cells were stimulated with 50 ng/ml PMA (Sigma) and 500 ng/ml ionomycin (Sigma), and treated with GolgiStop (BD Biosciences) for 5 h at 37°C. For intracellular staining, cells were fixed and permeabilized with the BD Cytofix/Cytoperm Fixation/Permeabilization Solution Kit (BD Biosciences), then stained with Ab for 30 min at 4°C. A BD LSR II flow cytometer was used for the analysis. A fluorescence activated cell sorting (FACS) Aria flow cytometer was used for cell sorting. Antibodies were prepared according to the concentration of different flow cytometry antibody instructions. The isotype control of the flow cytometry antibody was set and incubated at 4°C for 20 min in the dark and detected with a flow cytometer. For samples that require intracellular staining, a 2 μl/ml cell activation cocktail was added to stimulate the cells (Biolegend) and, incubated in a 37°C incubator for 4 h. Flow cytometry was used for detection. FlowJoV10 flow analysis software was applied for data analysis.

### 2.13 Statistical analysis

Prism software (GraphPad) was used for all statistical analyses, showed in each figure legend. Grouped data are expressed as mean ± SEM.

## 3 RESULTS

### 3.1 Decreased SPRY1 in psoriasis lesional skin and cultured keratinocytes

**FIGURE 1:**
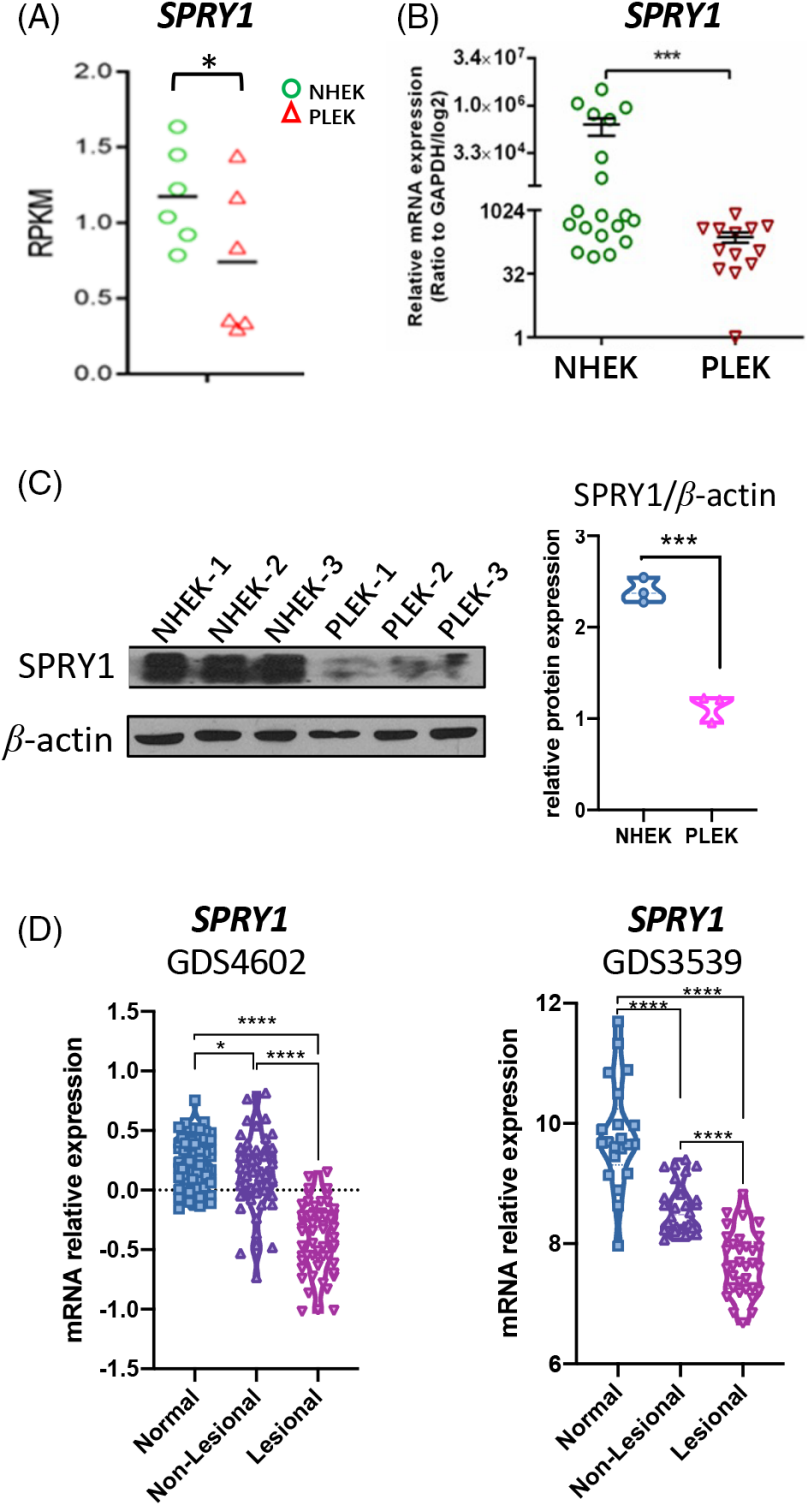
Decreased SPRY1 in psoriasis lesional skin and cultured keratinocytes. (A–C) The relative level of SPRY1 between NHEK and PLEK determined by RNAseq (A), qRT‐PCR (B) and western blot (C, ratio with *β*‐actin). (D) Re‐analysed SPRY1 expression level in psoriasis transcriptional datasets from GEO (datasets: GDS4602, GDS3539). NELK, human normal epidermal keratinocyte; PLEK, human psoriatic epidermal keratinocyte (statistical analyses by the mean of unpaired nonparametric *t*‐test, Mann–Whitney test)

To identify the key factors involved in psoriasis progression in keratinocytes, human epidermal keratinocytes were isolated from six normal and six psoriatic lesional skin samples and cultured in EpiGRO™ Human Epidermal Keratinocyte Complete Culture Media. RNA‐seq was performed on PLEKs) and NHEKs. Interestingly, the mRNA expression of SPRY1 was decreased in PLEKs (Figure 1A), which was confirmed in other PLEKs (*n* = 13) by qRT‐PCR (Figure 1B). The protein level of SPRY1 in PLEKs was also significantly decreased compared with that in NHEKs (Figure 1C). In summary, SPRY1 was decreased in PLEKs at both the mRNA and protein levels.

To further confirm these results, we analysed published datasets of human psoriatic skin in the GEO. The mRNA level of SPRY1 was repressed in nonlesional skin and markedly repressed in lesional human psoriatic skin (Gudjonsson et al., GDS4602/GSE13355; Higgs et al., GDS3539/GSE14905, Figure 1). These data indicate that SPRY1 expression is repressed in psoriatic lesional skin.

### 3.2 SPRY1 decreased in psoriasis epidermis and recovered after effective treatments

**FIGURE 2:**
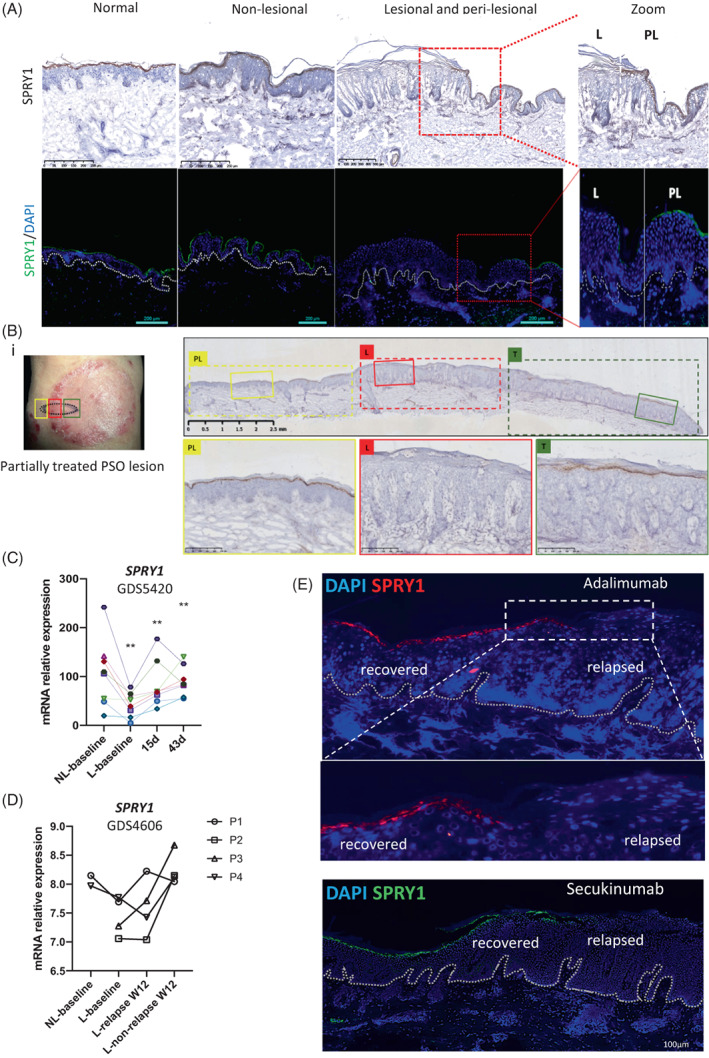
Dynamical alteration of SPRY1 in psoriasis epidermis. (A,B,E) IF and IHC detected the expression of SPRY1 in human normal skin from healthy donor, non‐lesional, peri‐lesional and lesional from psoriatic skin (A), and treated recovering skin by topical calcipotriol (B), adalimumab and secukinumab (E) from psoriasis patients. (C,D) Re‐analysed SPRY1 expression level in psoriasis transcriptional datasets from GEO, GDS5420 (C; from 8 individual patients) and GDS4606 (D; from four individual patients, P1‐4) (statistical analyses by the mean of paired *t*‐test)

Next, SPRY1 was examined by both immunohistochemistry and immunofluorescence staining in normal and psoriatic nonlesional, perilesional and lesional skin. SPRY1 was easily detectable in the granular layer of the normal epidermis and nonlesional and perilesional psoriatic epidermis but was absent or decreased in the psoriatic lesional epidermis (Figure 2A).

Then, we procured and immunostained a psoriatic plaque that had been treated with topical calcipotriol cream, which is an effective agent for psoriasis treatment, in the central zone, while the peripheral zone was left untreated. As shown in Figure 2B, deep immunostaining of SPRY1 was observed in the central recovery and perilesional epidermal granular layer but was absent in the untreated lesional epidermis. These data suggest that SPRY1 was dynamically expressed in different stages of the psoriatic epidermis (nonlesional, perilesional, lesional and recovered epidermis), correlating with cutaneous inflammation.

Accordingly, epidermal SPRY1 may be an early indicator of psoriatic inflammation, which may be involved in the pathogenesis of psoriasis initiation, recovery and relapse.

To further confirm this hypothesis, we analysed the RNA‐seq data in the GEO database, which is derived from psoriasis patients treated with brodalumab, an IL‐17A receptor antibody. Consistent with our results, the transcriptional level of SPRY1 was significantly downregulated in psoriatic lesional skin compared to nonlesional skin (*p* < 0.01). This expression was quickly reversed by brodalumab after 15 days and was maintained at a stable level for 43 days (*p* < 0.01, paired *t*‐test; Figure 2C, GDS5420). Since topical and systemic therapies could induce SPRY1 to reappear in the recovered psoriatic epidermis, we examined SPRY1 in relapsed skin from moderate‐to‐severe psoriasis patients treated with efalizumab (anti‐CD11a, Raptiva, from GDS4606, *n* = 4), adalimumab (anti‐tumour necrosis factor‐α [TNFα], Humira, our patient) and secukinumab (anti‐IL17, Cosentyx, our patient). Specifically, 12 weeks after ceasing efalizumab, SPRY1 mRNA was expressed in nonrelapsed psoriatic skin but was low in relapsed skin and nearly equal to the baseline level (Figure 2D, GDS4606). Similarly, SPRY1 was nearly absent from the relapsed epidermis but showed bright staining in the nonrelapsed epidermal granular layer of psoriatic patients treated with adalimumab and secukinumab (Figure 2E). Together, epidermal SPRY1 was dynamically expressed during psoriasis initiation, recovery and relapse, which may be involved in psoriasis pathogenesis.

### 3.3 Keratinocytes overexpressed SPRY1 alleviated IMQ‐induced psoriasis‐like skin inflammation

**FIGURE 3:**
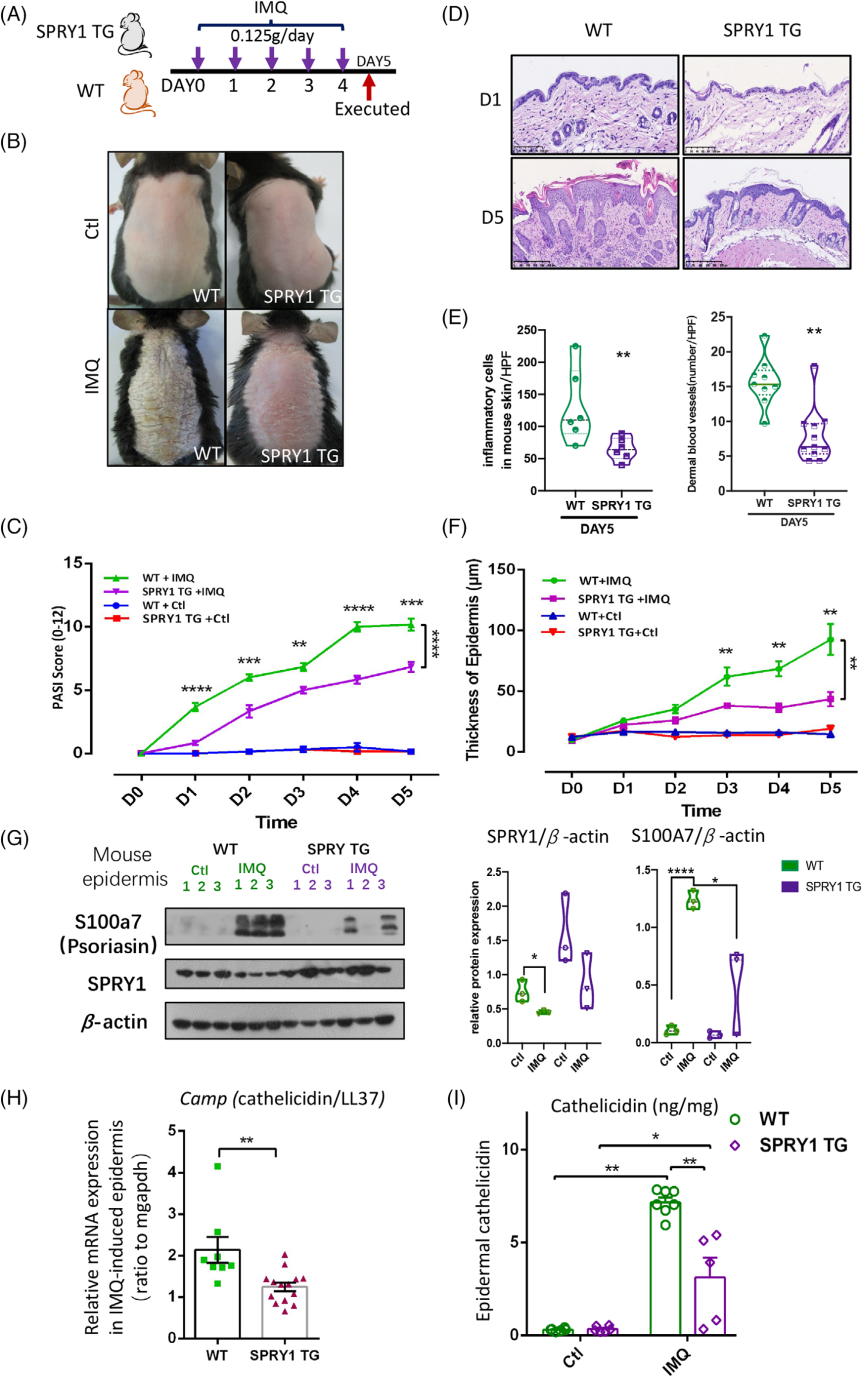
Keratinocytes overexpressed SPRY1 alleviated IMQ‐induced psoriasis‐form skin inflammation. (A) The workflow of IMQ‐induced mouse psoriasis‐form inflammation on Krt14‐SPRY1 transgenic (SPRY1 TG) and wide type mouse, vehicle as control (Ctl). (B–D) The phenotype, daily PASI score and Haematoxylin–eosin staining of IMQ‐induced mouse skin. (E) The number of inflammatory cells in mouse skin, and the number of dermal blood vessels per HPF. (F) Thickness of mouse back skin. (G) Relative expression level of psoriasin and SPRY1 in mouse epidermis before and after IMQ‐induced (*n* = 3). (H,I) mRNA and protein level of LL37 (*camp*) in mouse epidermis detected by qPCR and ELISA. HPF, high power field (C,F, paired *t*‐test; E,G,H, unpaired nonparametric *t*‐test, Mann–Whitney test)

It is well known that IMQ, a TLR7/8 ligand and potent immune activator, can induce psoriasis‐like skin inflammation.^15^, ^16^ Therefore, IMQ was used to assess the role of SPRY1 in the development of psoriasis. K14‐SPRY1 transgenic (Krt14‐SPRY1‐tg, STG) mice were developed as previously described,^12^ and daily topical application of IMQ was used on the back skin for five consecutive days (Figure 3A). Psoriasis‐like skin inflammation, local Psoriasis Area Severity Index (PASI) scores, histology and the course of skin inflammation were observed. The IMQ‐induced psoriasis‐like skin phenotype and local PASI, including erythema, scaling and induration, were considerably reduced in the STG mice compared with WT mice from days 1 to 5 of IMQ induction (Figure 3B,C). Furthermore, on day 5 of topical IMQ administration, epidermal thickness infiltrated dermal inflammatory cells and dermal blood vessels were reduced in STG mice compared with WT mice (Figure 3D,E). Significant differences in local PASI and epidermal thickness were observed between STG and WT mice (Figure 3C,F, paired *t*‐test).

Psoriasin, also known as S100a7, is a psoriasis‐specific antimicrobial peptides (AMP) and was upregulated in the IMQ‐induced mouse epidermis (Figure 3G). In contrast, S100a7 was greatly inhibited in the epidermis of STG mice (Figure 3G). In addition, IMQ significantly inhibited the expression of SPRY1 in WT mice (*p* < 0.05, Figure 3G). Cathelicidin, as known as LL37, another AMP produced by keratinocytes, which is encoded by the *CAMP* gene in humans, can form DNA‐LL37 complexes that stimulate plasmacytoid dendritic cells to secrete interferon α, which then activates myeloid dendritic cells, and activated myeloid dendritic cells secrete IL‐12 and IL‐23.^5^, ^6^, ^17^ Together, these AMPs, cytokines and immune cells were considered to initiate psoriasis. Cathelicidin plays an important role in the initiation of psoriasis and that was decreased in the epidermis of STG mice compared with WT mice after IMQ treatment (Figure 3H). Moreover, cathelicidin was upregulated at the protein level in the IMQ‐induced mouse epidermis, while this upregulation was significantly inhibited in the epidermis of STG mice (Figure 3I). These results indicate that SPRY1 alleviates IMQ‐induced psoriasis‐like skin lesions by inhibiting cathelicidin, which plays a critical role in the pathogenesis of psoriasis.^18^

### 3.4 Keratinocyte derived SPRY1 alleviated IMQ‐induced cutaneous inflammation

**FIGURE 4:**
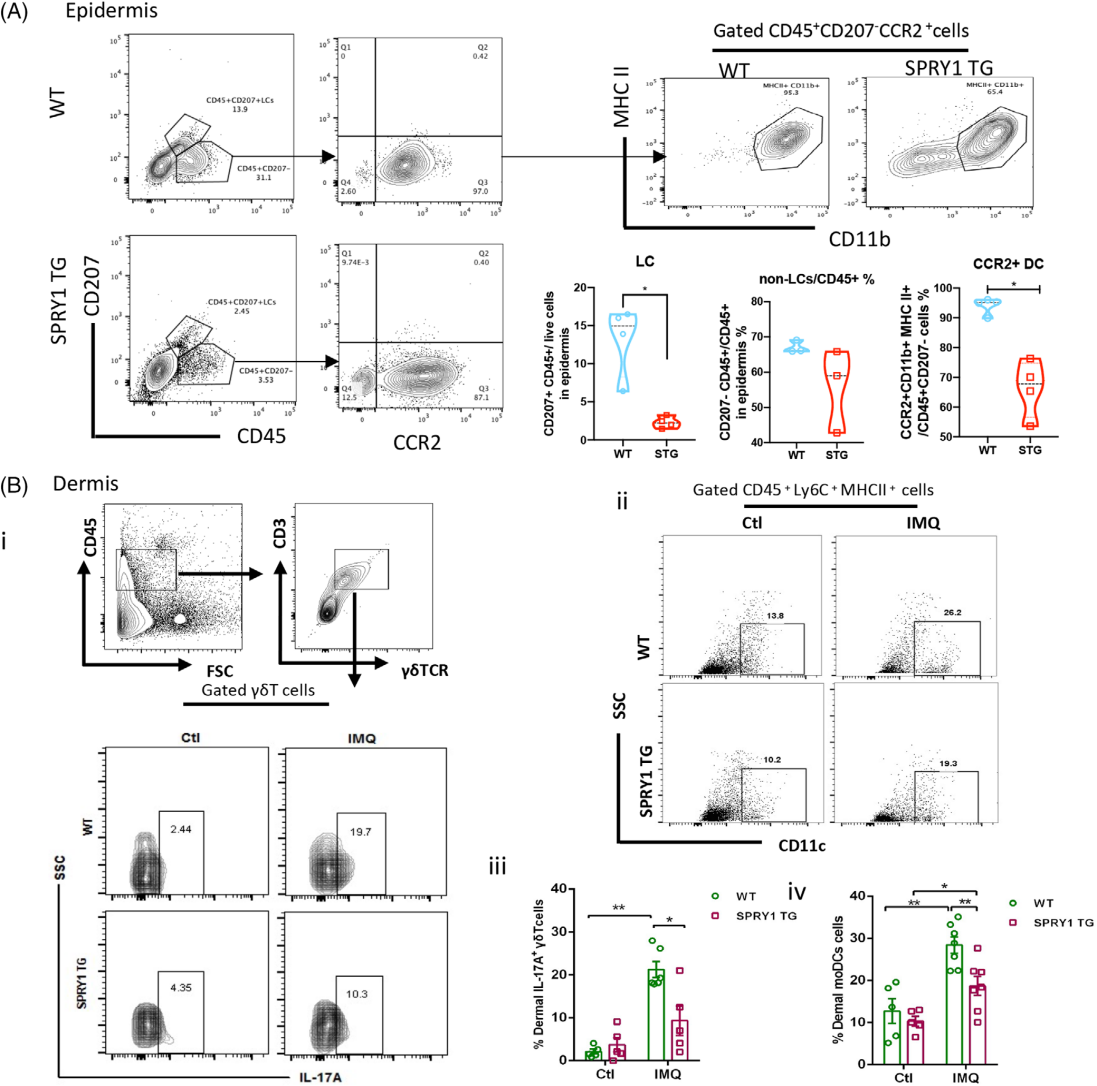
Keratinocyte derived SPRY1 alleviated IMQ‐induced skin inflammation. (A) CD45 + CD207 + Langerhans cells, CD45 + CD207− non‐Langerhans cells, and CD45 + CD207‐MHC II + CD11b + CCR2 + DC proportion in IMQ‐induced mouse epidermis through FACS (*n* ≥ 3). (B) Percentage of IL‐17A+ γδT/γδT, and Ly6C+ CD11c moDC/Ly6C+ monocytes in IMQ‐induced mouse dermis through FACS (*n* ≥ 5) (unpaired nonparametric *t*‐test, Mann–Whitney test)

Typically, during the immunopathogenesis of psoriasis, injured epidermal keratinocytes can release LL37 and S100A7, which activate DCs and initiate subsequent immune cell recruitment and interleukin (IL)‐23/Th17 cell axis inflammatory cascades.^6^, ^19^ Therefore, we examined the distribution of γδT cells and dendritic cells in the epidermis and dermis of IMQ‐induced mice by flow cytometry. As shown in Figure 4A, Langerhans cells (LCs, CD45^+^CD207^+^ cells) and non‐LCs/CD45^+^ cells, especially CCR2^+^ DCs, were significantly decreased in the epidermis of K14‐SPRY1 tg mice after IMQ treatment. T cell activation is a key process in psoriasis development.^20^ Dermal IL‐17A‐producing γδT cells and the percentage of dermal moDCs (CD45^+^Ly6c^+^ major histocompatibility complex class II (MHCII^+^)CD11c^+^) were reduced in the epidermis of STG mice compared with WT mice (Figure 4B). These data indicated that SPRY1 in keratinocytes inhibited γδT cells and DCs, which are crucial components of the cutaneous innate immune system.^21^, ^22^

### 3.5 SPRY1 protect psoriatic cutaneous inflammation by regulating epidermal cathelicidin

**FIGURE 5:**
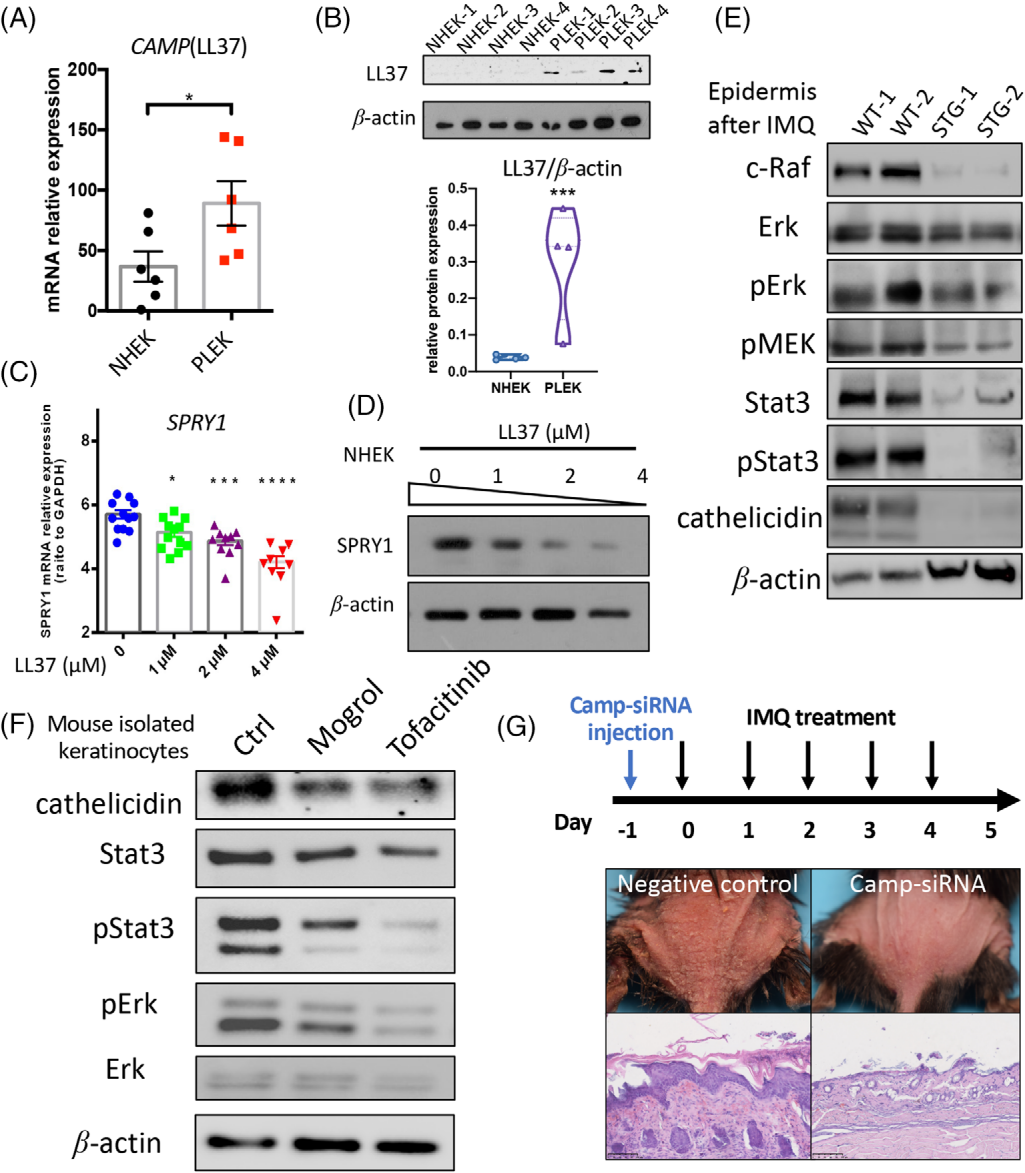
SPRY1 protect psoriatic cutaneous inflammation mediated by interaction with epidermal cathelicidin. (A,B) Relative mRNA (*n* = 6) and protein (*n* = 4) level of LL37 in cultured NHEK and PLEK. (C,D) SPRY1 expression after LL37 treatment in protein and mRNA level. E, Western blots shown mitogen‐activated protein kinase (MAPK) pathway, Stat3 and cathelicidin expression in IMQ‐induced mouse epidermis (*n* = 2). (F) Western blot detected cathelicidin level in cultured WT mouse keratinocytes after Stat3 inhibitors, mogrol (10 μM) and tofacitinib (1 nM). (G) The workflow and mouse phenotype of Camp‐siRNA subcutaneous injection and IMQ treatment (*n* = 3). WT, wild type; STG, K14‐SPRY1 transgenic mice (unpaired nonparametric *t*‐test, Mann–Whitney test)

Since SPRY1 plays a protective role in IMQ‐induced psoriasis‐like skin inflammation, we investigated how SPRY1 regulated skin inflammation in human epidermal keratinocytes. Previous reports have demonstrated that the activation of LL37 induces moDCs in psoriasis.^6^ Here, we confirmed that LL37 was much higher in PLEKs than in NHEKs at both the mRNA and protein levels (Figure 5A,B). Intriguingly, the mRNA and protein expression of SPRY1 in NHEKs was dose‐dependently decreased by 1–4 μM LL37 treatment (Figure 5C,D). Therefore, based on the results of Krt14‐SPRY1‐tg mice, which exhibited less level of cathelicidin in the epidermis than WT mice (Figure 3I), we may conclude that SPRY1 and LL37 are negatively and mutually regulated in psoriasis. This effect may be the reason behind the high SPRY1 and low LL37 expression in the normal epidermis, while oppositely high LL37 and low SPRY1 expression have been observed in the psoriatic epidermis.

We further screened signalling pathways that may be associated with SPRY1. SPRY1 is an antagonist in the RTK/MAPK signalling pathway. In the IMQ‐induced mouse epidermis, cathelicidin was inhibited by SPRY1 overexpression, which was accompanied by decreased levels of c‐Raf/pMek/pErk and pStat3 (Figure 5E). As reported, the promoter sequence of *CAMP*, which encodes LL37, has binding sites for STAT3.^23^ To further confirm the regulation of Stat3 and cathelicidin, isolated WT mouse keratinocytes were cultured with Stat3 inhibitors (mogrol and tofacitinib), and cathelicidin was decreased in keratinocytes (Figure 5F). Moreover, cathelicidin knockdown by subcutaneous injection of 2′‐oMe modified siRNA, alleviated IMQ‐induced cutaneous inflammation (Figure 5G). Taken together, these data indicate that SPRY1 may inhibit cathelicidin through MAPK and Stat3 signalling.

## 4 DISCUSSION

Cutaneous AMPs and immune cell infiltration not only represent skin inflammation but also play key roles in the initiation of skin disease. For instance, specific AMPs like cathelicidin/LL37, psoriasin (S100A7) and inflammatory cells like moDCs were crucial initial events of psoriasis.^5^, ^6^, ^17^ When knocked down cathelicidin in a mouse, IMQ‐induced psoriasis‐like skin inflammation was hardly developed (Figure 5G). So, LL37 is considered an important initial molecule in psoriasis. In vivo, utilizing epidermis‐specific Krt14‐SPRY1 transgenic mice, LL37 decreased significantly when Spry1 epidermis‐specific overexpressed, in a psoriasis‐like mouse model. The promoter sequence of *CAMP*, which encodes LL37, has binding sites for STAT3.^23^ And we verified that Stat3 inhibitors can suppress the production of cathelicidin in keratinocytes (Figure 5F). Activation of the STAT3 is important in psoriasis pathogenesis^24^, ^25^ so that both topical and systemic JAK/STAT inhibitors have been emerging and promising in psoriasis therapy.^26^, ^27^, ^28^ Stat3 activation was suppressed in IMQ‐induced epidermis when sprouty1 was overexpressed (Figure 5E). Those data indicated sprouty1 can inhibit LL37 by suppressing Stat3 activation in psoriasis.

Meanwhile, sprouty1 in was decreased after LL37 treatment dose‐dependently in vitro (Figure 5C,D). The feedback regulation relationship between sprouty1 and LL37 may amplify psoriasis cutaneous inflammation and affect the pathogenesis of psoriasis.

Surprisingly, keratinocyte‐derived sprouty1 not only inhibited epidermal LCs and DCs but also suppressed the recruitment of dermal γδT cells and moDC infiltration (Figure 4). The role of T cells must be concerned, including CD4+ T cells, tissue‐resident memory CD8+ T cells and regulatory T cells,^29^, ^30^, ^31^ as well as DCs.^32^ As reported, some studies showed that sprouty1 could influence the survival and exhaustion of T cells.^13^, ^14^ Recently, Bruton's tyrosine kinase inhibitors were reported could decrease the production of IL‐23 and TNF‐α from CD11c + DCs, as well as IL‐17A from γδ + T cells in IMQ‐induced psoriatic skin inflammation.^33^ The limitations of this study are including that do not mention the function of sprouty1 in psoriasis T cells and the mechanism of effect between sprouty1 and DCs, which will be continued in‐depth studies in the near future.

In conclusion, our data indicated the regulation between sprouty1 and LL37 that might link keratinocytes and cutaneous innate immune in psoriasis. This study provides new evidence for the pathogenesis of psoriatic keratinocytes. Sprouty1 plays a preventive role in psoriatic skin inflammation, which is a possible novel therapeutic target.

## AUTHOR CONTRIBUTIONS

Yuan Zhou and Ping Wang conducted the experiments, and wrote the paper; Xue‐Yan Chen, Bing‐Xi Yan and Fan Xu documented and analysed data; Lilla Landeck and Zhao‐Yuan Wang modified the paper; Xiao‐Yong Man designed the experiments; Xiao‐Yong Man and Min Zheng supervised methods, administrated and fund the project. Yuan Zhou and Ping Wang contributed equally to this work.

## FUNDING INFORMATION

This study was supported by grants from the National Natural Science Foundation of China (No. 81930089, 81630082, 81773318, 82103709).

## CONFLICT OF INTEREST

The authors declare they have no conflicts of interest.

## Supporting information


**Table S1**. Patient and healthy control demographics.
**Table S2**. Antibodies used for IHC, IF, Western Blot and flow cytometry.

## Data Availability

The datasets analysed during the current study are available in the GEO repository. Open‐accessed published RNAseq datasets of human psoriatic skin from GEO, Gene Expression Omnibus GDS4602, GDS3539, GDS5420 and GDS4606, were referred to in this paper. These data were derived from the following resources available in the public domain: GDS4602: https://www.ncbi.nlm.nih.gov/sites/GDSbrowser?acc=GDS460; GDS3539: https://www.ncbi.nlm.nih.gov/sites/GDSbrowser?acc=GDS3539; GDS5420: https://www.ncbi.nlm.nih.gov/sites/GDSbrowser?acc=GDS5420; GDS4606: https://www.ncbi.nlm.nih.gov/sites/GDSbrowser?acc=GDS4606.

## References

[1] Boehncke WH , Schon MP . Psoriasis. Lancet. 2015;386(9997):983‐994. 26025581 10.1016/S0140-6736(14)61909-7

[2] Nestle FO , Kaplan DH , Barker J . Psoriasis. N Engl J Med. 2009;361(5):496‐509. 19641206 10.1056/NEJMra0804595

[3] Lowes MA , Bowcock AM , Krueger JG . Pathogenesis and therapy of psoriasis. Nature. 2007;445(7130):866‐873. 17314973 10.1038/nature05663

[4] Griffiths CE , Barker JN . Pathogenesis and clinical features of psoriasis. Lancet. 2007;370(9583):263‐271. 17658397 10.1016/S0140-6736(07)61128-3

[5] Lowes MA , Suarez‐Farinas M , Krueger JG . Immunology of psoriasis. Annu Rev Immunol. 2014;32:227‐255. 24655295 10.1146/annurev-immunol-032713-120225 PMC4229247

[6] Kim J , Krueger JG . The immunopathogenesis of psoriasis. Dermatol Clin. 2015;33(1):13‐23. 25412780 10.1016/j.det.2014.09.002

[7] Tschachler E . Psoriasis: the epidermal component. Clin Dermatol. 2007;25(6):589‐595. 18021897 10.1016/j.clindermatol.2007.09.021

[8] Nadeem A , Ahmad SF , Al‐Harbi NO , El‐Sherbeeny AM , Al‐Harbi MM , Almukhlafi TS . GPR43 activation enhances psoriasis‐like inflammation through epidermal upregulation of IL‐6 and dual oxidase 2 signaling in a murine model. Cell Signal. 2017;33:59‐68. 28212864 10.1016/j.cellsig.2017.02.014

[9] Nadeem A , Al‐Harbi NO , Ansari MA , et al. Psoriatic inflammation enhances allergic airway inflammation through IL‐23/STAT3 signaling in a murine model. Biochem Pharmacol. 2017;124:69‐82. 27984001 10.1016/j.bcp.2016.10.012

[10] Calautti E , Avalle L , Poli V . Psoriasis: a STAT3‐centric view. Int J Mol Sci. 2018;19(1):171. doi:10.3390/ijms19010171 PMC5796120 29316631

[11] Ahmad SF , Ansari MA , Nadeem A , et al. Resveratrol attenuates pro‐inflammatory cytokines and activation of JAK1‐STAT3 in BTBR T(+) Itpr3(tf)/J autistic mice. Eur J Pharmacol. 2018;829:70‐78. 29654783 10.1016/j.ejphar.2018.04.008

[12] Wang P , Zhou Y , Yang JQ , et al. The role of Sprouty1 in the proliferation, differentiation and apoptosis of epidermal keratinocytes. Cell Prolif. 2018;51(5):e12477. 30039569 10.1111/cpr.12477 PMC6528922

[13] Shehata HM , Khan S , Chen E , Fields PE , Flavell RA , Sanjabi S . Lack of Sprouty 1 and 2 enhances survival of effector CD8(+) T cells and yields more protective memory cells. Proc Natl Acad Sci U S A. 2018;115(38):E8939‐E8947. 30126987 10.1073/pnas.1808320115 PMC6156615

[14] Chen QY , Li YN , Wang XY , et al. Tumor fibroblast‐derived FGF2 regulates expression of SPRY1 in esophageal tumor‐infiltrating T cells and plays a role in T‐cell exhaustion. Cancer Res. 2020;80(24):5583‐5596. 33093168 10.1158/0008-5472.CAN-20-1542

[15] Zhou Y , Wang P , Yan BX , et al. Quantitative proteomic profile of psoriatic epidermis identifies OAS2 as a novel biomarker for disease activity. Front Immunol. 2020;11:1432. 32849499 10.3389/fimmu.2020.01432 PMC7410923

[16] van der Fits L , Mourits S , Voerman JS , et al. Imiquimod‐induced psoriasis‐like skin inflammation in mice is mediated via the IL‐23/IL‐17 axis. J Immunol. 2009;182(9):5836‐5845. 19380832 10.4049/jimmunol.0802999

[17] Armstrong AW , Read C . Pathophysiology, clinical presentation, and treatment of psoriasis: a review. JAMA. 2020;323(19):1945‐1960. 32427307 10.1001/jama.2020.4006

[18] Takahashi T , Yamasaki K . Psoriasis and antimicrobial peptides. Int J Mol Sci. 2020;21(18):6791. doi:10.3390/ijms21186791 PMC7555190 32947991

[19] Gaffen SL , Jain R , Garg AV , Cua DJ . The IL‐23‐IL‐17 immune axis: from mechanisms to therapeutic testing. Nat Rev Immunol. 2014;14(9):585‐600. 25145755 10.1038/nri3707 PMC4281037

[20] Karczewski J , Dobrowolska A , Rychlewska‐Hanczewska A , Adamski Z . New insights into the role of T cells in pathogenesis of psoriasis and psoriatic arthritis. Autoimmunity. 2016;49(7):435‐450. 27050731 10.3109/08916934.2016.1166214

[21] Chen YL , Hardman CS , Yadava K , Ogg G . Innate lymphocyte mechanisms in skin diseases. Annu Rev Immunol. 2020;38:171‐202. 32340577 10.1146/annurev-immunol-082919-093554

[22] Kashem SW , Haniffa M , Kaplan DH . Antigen‐presenting cells in the skin. Annu Rev Immunol. 2017;35:469‐499. 28226228 10.1146/annurev-immunol-051116-052215

[23] Miraglia E , Nylen F , Johansson K , et al. Entinostat up‐regulates the CAMP gene encoding LL‐37 via activation of STAT3 and HIF‐1alpha transcription factors. Sci Rep. 2016;6:33274. 27633343 10.1038/srep33274 PMC5025742

[24] Kishimoto M , Komine M , Sashikawa‐Kimura M , et al. STAT3 activation in psoriasis and cancers. Diagnostics. 2021;11(10):1903. doi:10.3390/diagnostics11101903 PMC8534757 34679602

[25] Ravipati A , Nolan S , Alphonse M , et al. IL‐6R/signal transducer and activator of transcription 3 signaling in keratinocytes rather than in T cells induces psoriasis‐like dermatitis in mice. J Invest Dermatol. 2021;142:1126‐1135.e4. 34626614 10.1016/j.jid.2021.09.012 PMC8957489

[26] Fragoulis GE , McInnes IB , Siebert S . JAK‐inhibitors. New players in the field of immune‐mediated diseases, beyond rheumatoid arthritis. Rheumatology. 2019;58(Suppl 1):i43‐i54. 30806709 10.1093/rheumatology/key276 PMC6390879

[27] Nogueira M , Puig L , Torres T . JAK inhibitors for treatment of psoriasis: focus on selective TYK2 inhibitors. Drugs. 2020;80(4):341‐352. 32020553 10.1007/s40265-020-01261-8

[28] Solimani F , Meier K , Ghoreschi K . Emerging topical and systemic JAK inhibitors in dermatology. Front Immunol. 2019;10:2847. 31849996 10.3389/fimmu.2019.02847 PMC6901833

[29] Hawkes JE , Chan TC , Krueger JG . Psoriasis pathogenesis and the development of novel targeted immune therapies. J Allergy Clin Immunol. 2017;140(3):645‐653. 28887948 10.1016/j.jaci.2017.07.004 PMC5600287

[30] Nussbaum L , Chen YL , Ogg GS . Role of regulatory T cells in psoriasis pathogenesis and treatment. Br J Dermatol. 2021;184(1):14‐24. 32628773 10.1111/bjd.19380

[31] Leijten EF , van Kempen TS , Olde Nordkamp MA , et al. Tissue‐resident memory CD8+ T cells from skin differentiate psoriatic arthritis from psoriasis. Arthritis Rheumatol. 2021;73(7):1220‐1232. 33452865 10.1002/art.41652 PMC8362143

[32] Al‐Harbi NO , Nadeem A , Ahmad SF , et al. Therapeutic treatment with Ibrutinib attenuates imiquimod‐induced psoriasis‐like inflammation in mice through downregulation of oxidative and inflammatory mediators in neutrophils and dendritic cells. Eur J Pharmacol. 2020;877:173088. 32234429 10.1016/j.ejphar.2020.173088

[33] Nadeem A , Ahmad SF , Al‐Harbi NO , et al. Bruton's tyrosine kinase inhibitor suppresses imiquimod‐induced psoriasis‐like inflammation in mice through regulation of IL‐23/IL‐17A in innate immune cells. Int Immunopharmacol. 2020;80:106215. 31982823 10.1016/j.intimp.2020.106215

