# Supplementary material for: Sprouty1 exerts a preventive effect on the initiation of psoriasis by inhibiting innate immune antimicrobial peptide cathelicidin and immunocytes
Source: Cell Prolif. 2022 Jun 18;55(10):e13290. doi: 10.1111/cpr.13290 (PMC9528767; doi:10.1111/cpr.13290)
Supplement: Supplementary file 1 — Table S1. Patient and healthy control demographics. Table S2. Antibodies used for IHC, IF, Western Blot and flow cytometry. [file CPR-55-e13290-s001.docx]

**Supplementary Tables:**

Supplementary Table 1. Patient and healthy control demographics

| patient | gender | age | PASI | Skin samples taken site |
| --- | --- | --- | --- | --- |
| Psoriasis | | | | |
| PSO-1 | Male | 40 | 40.4 | Back |
| PSO-2 | Male | 65 | 25 | Abdomen |
| PSO-3 | Female | 61 | 31.4 | Arm |
| PSO-4 | Male | 43 | 15 | Arm |
| PSO-5 | Male | 51 | 12.5 | Leg |
| Psoriasis after biological treatment | | | | |
| PSO-Adalimumab | Male | 50 | 20 | Back |
| PSO-Secukinumab | Male | 52 | 28 | Leg |
| Healthy | | | | |
| HC-1 | Male | 62 |  | Arm |
| HC-2 | Female | 56 |  | Leg |
| HC-3 | Male | 53 |  | Back |
| HC-4 | Male | 37 |  | Abdomen |
| HC-5 | Female | 34 |  | Back |

Supplementary Table 2. Antibodies used for IHC, IF, WB and flow cytometry

| Antigen | Supplier | Clone | Isotype | Molecular Weight | Catalogue number | Dilution |
| --- | --- | --- | --- | --- | --- | --- |
| Sprouty1 | abcam | corresponding to AA1-111 of Human Sprouty 1 | Mouse IgG2a | 37kDa | ab56670 | 1:200 for IHC |
|  |  |  |  |  |  | 1:200 for IF |
| Sprouty1 | abcam | corresponding to Human Sprouty 1/Spry-1 aa 100-200 | Rabbit polyAb |  | ab111523 | 1:1000 for WB |
| cathelicidin/LL37 | abcam | corresponding to Human Cathelicidin/CLP aa 31-170. | Rabbit IgG | / | ab180760 | 1:200 for IF |
| cathelicidin/LL37 | santa | raised against amino acids 131-170 | Mouse IgG1 kappa | 7-17kDa | sc-166770 | 1:1000 for WB |
| *β*-actin | CST | 13E5 | Rabbit mAb | 45kDa | #4970 | 1:1000 for WB |
| S100a7/Psoriasin | abcam | Recombinant full length protein corresponding to Human Psoriasin | Mouse IgG1 kappa | 11kDa | ab13680 | 1:1000 for WB |
| c-Raf | CST | D4B3J | Rabbit mAb IgG | 75kDa | #53745 | 1:1000 for WB |
| Erk | CST | 137F5 | Rabbit mAb | 42/44kDa | #4695 | 1:1000 for WB |
| pErk | CST | Thr202/Tyr204(D13.14.4E) | Rabbit mAb | 42/44kDa | #4370 | 1:1000 for WB |
| pMEK | CST | Ser217/221(41G9) | Rabbit mAb | 45kDa | #9154 | 1:1000 for WB |
| Stat3 | CST | 79D7 | Rabbit mAb | 79/86kDa | #4904 | 1:1000 for WB |
| pStat3 | CST | Tyr705 (D3A7) | Rabbit mAb | 79/86kDa | #9145 | 1:1000 for WB |
|  | | | | | | |
| anti-mouse CD45 | biolegend | 30-F11 | Rat IgG2b, κ | / | Cat#103138 | 0.5ug/10^6^cells for flow |
| anti-mouse MHC-II | biolegend | M5/114.15.2 | Rat IgG2b, κ | / | Cat#107605 | 0.25ug/10^6^cells for flow |
| anti-mouse | biolegend | 4C7 | Mouse IgG2a, κ | / | Cat#144203 | 1.0ug/10^6^cells for flow |
| CD207 |  |  |  |  |  |  |
| anti-mouse | biolegend | SA203G11 | Rat IgG2b, κ | / | Cat#150621 | 0.25ug/10^6^cells for flow |
| CCR2 |  |  |  |  |  |  |
| anti-mouse | biolegend | M1/70 | Rat IgG2b, κ | / | Cat#101261 | 0.25ug/10^6^cells for flow |
| CD11b |  |  |  |  |  |  |
